# The novel circCLK3/miR-320a/FoxM1 axis promotes cervical cancer progression

**DOI:** 10.1038/s41419-019-2183-z

**Published:** 2019-12-12

**Authors:** Hanqing Hong, Hai Zhu, Shujun Zhao, Kaili Wang, Nan Zhang, Yun Tian, Yan Li, Yaping Wang, Xiaofeng Lv, Tianxiang Wei, Yan Liu, Suzhen Fan, Yang Liu, Yuan Li, Aojie Cai, Shuo Jin, Qiaohong Qin, Hongyu Li

**Affiliations:** 1 Department of obstetrics and gynecology, The Third Affiliated Hospital of Zhengzhou University, 450052 Zhengzhou, China; 2 Zhengzhou Key Laboratory of Gynecological Oncology, 450052 Zhengzhou, China; 3 Department of medical genetics and cell biology, School of Basic Medical Sciences of Zhengzhou University, 450052 Zhengzhou, China; 4 Department of Obstetrics and Gynecology, The First Affiliated Hospital of Zhengzhou University, 450052 Zhengzhou, China; 5 Genetics and prenatal diagnosis center, The First Affiliated Hospital of Zhengzhou University, 450052 Zhengzhou, China

**Keywords:** Non-coding RNAs, Oncogenesis

## Abstract

As a new class of non-coding RNA, circular RNAs (circRNAs) play crucial roles in the development and progression of various cancers. However, the detailed functions of circRNAs in cervical cancer have seldom been reported. In this study, circRNA sequence was applied to detect the differentially expressed circRNAs between cervical cancer tissues and adjacent normal tissues. The relationships between circCLK3 level with clinicopathological characteristics and prognosis were analyzed. In vitro CCK-8, cell count, cell colony, cell wound healing, transwell migration and invasion, and in vivo tumorigenesis and lung metastasis models were performed to evaluate the functions of circCLK3. The pull-down, RNA immunoprecipitation (RIP), luciferase reporter and rescue assays were employed to clarify the interaction between circCLK3 and miR-320a and the regulation of miR-320a on FoxM1. We found that the level of circCLK3 was remarkably higher in cervical cancer tissues than in adjacent normal tissues, and closely associated with tumor differentiation, FIGO stage and depth of stromal invasion. Down-regulated circCLK3 evidently inhibited cell growth and metastasis of cervical cancer in vitro and in vivo, while up-regulated circCLK3 significantly promoted cell growth and metastasis in vitro and in vivo. The pull-down, luciferase reporter and RIP assays demonstrated that circCLK3 directly bound to and sponge miR-320a. MiR-320a suppressed the expression of FoxM1 through directly binding to 3′UTR of FoxM1 mRNA. In addition, FoxM1 promoted cell proliferation, migration, and invasion of cervical cancer, while miR-320a suppressed cell proliferation, migration, and invasion through suppressing FoxM1, and circCLK3 enhanced cell proliferation, migration and invasion through sponging miR-320a and promoting FoxM1 expression. In summary, circCLK3 may serve as a novel diagnostic biomarker for disease progression and a promising molecular target for early diagnoses and treatments of cervical cancer.

## Introduction

Cervical cancer is the fourth most common cancer and major cause of cancer deaths for women worldwide^1,2^. With the widespread application of vaccination against human papilloma virus (HPV) and effective periodic cancer screening, a decreased incidence of cervical cancer and prompt surgical treatment in the early stages emerged. Nevertheless, cervical cancer still exhibits high invasion and mortality. However, the detailed mechanisms underlying progression of cervical cancer remain largely elusive. Therefore, it is essential to explore the precise mechanisms of cervical cancer progression, and seek brand new therapeutic targets.

In recent decade, non-coding RNA has become a hotspot in the field of tumors. As a novel type of non-coding RNA, circular RNAs (circRNAs) are attracting great attention in the field of genomic research in recent years. Compared with linear RNAs, circRNAs are characterized by covalently closed continuous loop with neither a 5′- and 3′- end nor a polyadenylated tail^3,4^, and more conservative and stable in cells. Currently, multiple mechanisms of circRNAs have been investigated, such as binding to RNA-binding protein (RBP)^5^, regulating splicing factor^6^ and encoding protein^7^. In 2011, Professor Pandolfi et al. threw a competing endogenous RNA (ceRNA) hypothesis that mRNA, long non-coding RNAs (lncRNAs), and pseudogenes could competitively bind to the microRNAs (miRNAs) to modulate gene expression^8^. Hansen et al. found two circRNAs, ciRS-7 and circular *Sry* RNA, and they first determined that both ciRS-7 and circular *Sry* RNA could act as ceRNAs by competitively binding to miR-7 or miR-138, respectively^9^. Thereafter, increasing mounting evidence demonstrated that circRNAs may act as ceRNAs by competitively binding to miRNAs and thus regulate downstream gene expression. However, the function of circRNAs in cervical cancer is rarely reported. In this study, circRNA sequencing between 3 paired fresh frozen cervical cancer tissues and matched normal tissues identified 118 differentially expressed circRNAs, including 82 up-regulated and 36 down-regulated circRNAs, with fold change >2 or <0.5, and *p* < 0.05. Of these up-regulated circRNAs, circCLK3, also named circ_0104541, was significantly higher in cervical cancer tissues than adjacent normal tissues, which was also identified by quantitative real-time PCR (qRT-PCR) results. Functionally, circCLK3 promoted cell proliferation, migration, and invasion. Furthermore, pull-down, luciferase reporter and RIP assay demonstrated that circCLK3 acted as a ceRNA to sponge miR-320a.

MiRNAs, 19–25 nucleotides in length, are the most important and most studied type of small non-coding RNA^10–12^. A great quantity of researches has demonstrated that miRNAs play important roles in the development and progression of various cancers. MiR-320a played an indispensable role in cell proliferation, migration, invasion, apoptosis, and chemosensitivity in multiple cancers, such as liver cancer^13^, salivary adenoid cystic carcinoma^13^, colorectal cancer^14^, myeloma^15^, and gastric cancer^16^. However, only one article reported the role of miR-320a in cervical cancer^17^. Accordingly, the detail biological functions and underlying molecular mechanisms of miR-320a in cervical cancer progression remain to be explored. In this study, molecular experiments indicated that miR-320a suppressed the expression of FoxM1 through directly binding to 3′UTR of FoxM1 mRNA, thereby inhibiting cell proliferation, migration, and invasion through in cervical cancer.

FoxM1, a typical transcription factor of Forkhead Box protein family, has been suggested to participate in various physiological processes of life^18–21^. FoxM1 has been reported to promote cell proliferation, migration, invasion, and EMT in a variety of human cancers^22–24^. As we all know, Ki-67 is a biomarker of cell proliferation, and Bcl-2 is a definite protein of anti-apoptosis. Wang et al. summarized that FoxM1 promoted cell proliferation of gastric cancer, and positively correlated with Ki-67 and Bcl-2 expression^25^. E-Cadherin, N-Cadherin, and Vimentin are the most important and common markers of EMT^26,27^. Low expression of E-Cadherin and high expression of N-Cadherin and Vimentin correspond to the process of EMT, while high expression of E-Cadherin and low expression of N-Cadherin and Vimentin indicate the process of mesenchymal-epithelial transition (MET). Zhang et al. concluded that FoxM1 promotes cell EMT by regulating E-Cadherin, Caveolin-1, urokinase-type plasminogen activator (uPA), and urokinase-type plasminogen activator receptor (uPAR)^28^. However, the molecular mechanisms underlying FoxM1 overexpression remain unclear. In a recent study, miR-320a promoted cell viability, migration, and invasion by directly targeting FoxM1^29^. In this study, we found that circCLK3 and FoxM1 both possess binding sites of miR-320a, and demonstrated that circCLK3 promotes the expression of FoxM1 by sponging miR-320a, forming a new theoretical basis for cervical cancer progression and creating a possible direction for targeted therapy.

## Results

### CircCLK3 is up-regulated in cervical cancer tissues and closely correlated with clinicopathological features

**Fig. 1:**
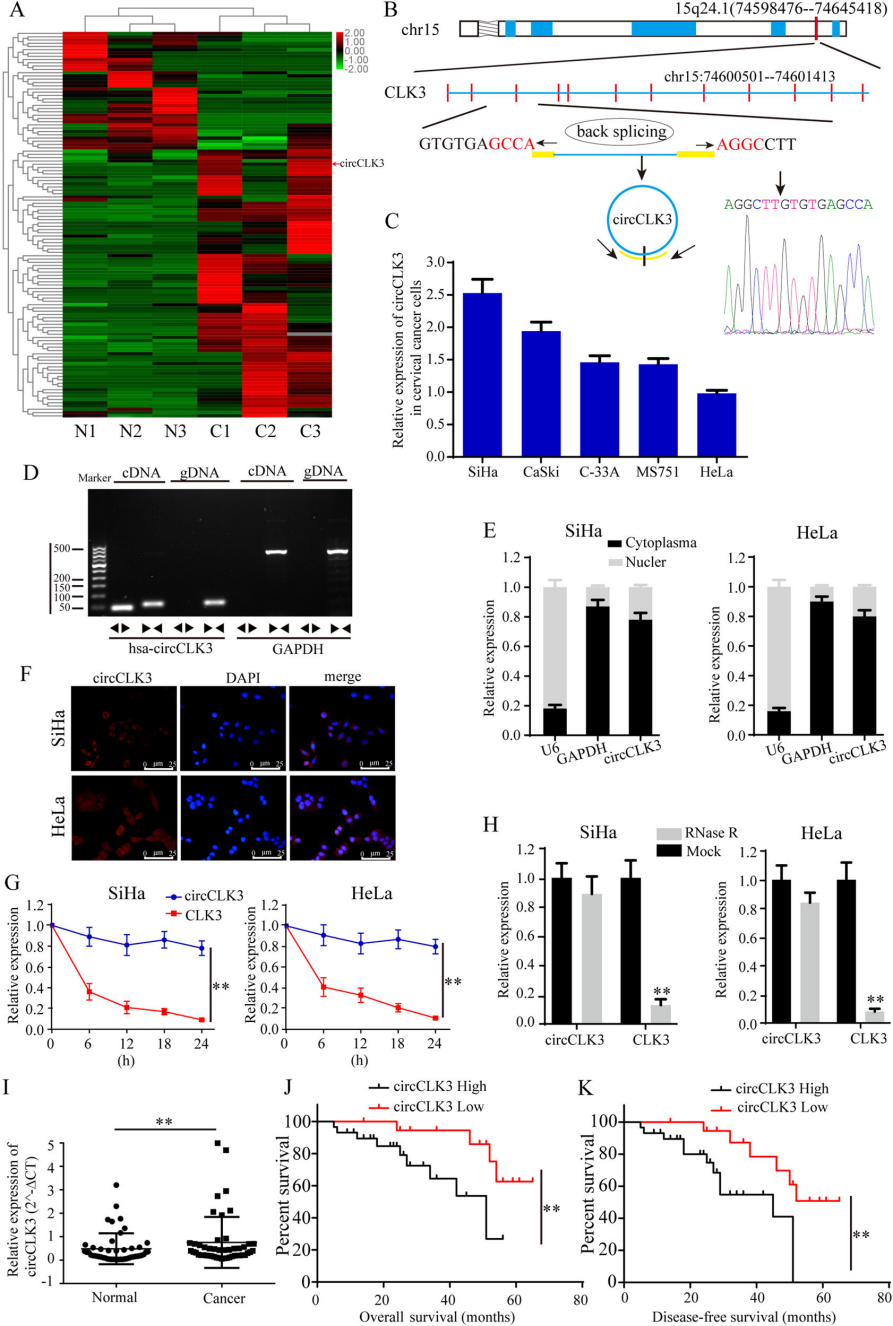
CircCLK3 expression is relatively highly expressed in cervical cancer tissues and predicts poor prognosis. **a** CircRNA sequencing was adapted to detect the differentially expressed circRNAs between cervical cancer tissues and matched normal tissues. **b** The genomic loci of the CLK3 gene and circCLK3. Sanger sequencing confirmed the head-to-hail splicing. **c** The expression level of circCLK3 in 5 cervical cancer cell lines relative to HeLa cell. **d** The gel electrophoresis validated the existence of circCLK3. Divergent primers amplified circCLK3 in cDNA but not gDNA. **e** qRT-PCR results of U6, GAPDH, and circCLK3 expressions in cell nuclei and cytoplasm in SiHa and HeLa cells. **f** RNA-FISH assays were performed to identify the subcellular location of circCLK3 with the cy3-labeled circCLK3 probe. The results showed that most of circCLK3 was located in cytoplasm in SiHa and HeLa cells. **g** qRT-PCR results of circCLK3 and CLK3 expression after treatment with actinomycin D at the indicated time points in SiHa and HeLa cells. **h** qRT-PCR results of circCLK3 and CLK3 expression after treatment with RNase R in SiHa and HeLa cells. **i** CircCLK3 expression was significantly higher in 48 paired fresh frozen cervical cancer tissues compared with adjacent normal tissues. **j**, **k** Kaplan–Meier Plotter analysis of the correlation between circCLK3 level with overall survive or disease-free survive of cervical cancer patients. Data are reported as means ± standard deviation of three independent experiments. *p < 0.05; **p < 0.01.

In order to seek for key circRNAs in the progression of cervical cancer, circRNA expression profiles were explored by circRNA sequencing between 3 paired fresh frozen cervical cancer tissues and matched normal tissues. A total of 118 differentially expressed circRNAs, including 82 up-regulated and 36 down-regulated circRNAs, was identified with fold change >2 or <0.5, and *p* < 0.05 (Fig. 1a). Among these up-regulated circRNAs, circ_0104541 was significantly overexpressed in cervical cancer tissues compared with adjacent normal tissues. According to the circbase database^30^ annotation, circ_0104541, which is an exonic type of circRNA spliced from CLK3 gene on chr15:74600501-74601413 and finally forming a sense-overlapping circular transcript of 913 nt (Fig. 1b), is named as circCLK3. Sanger sequencing confirmed the head-to-tail splicing (Fig. 1b). The levels of circCLK3 in 5 cervical cancer cell lines were detected. According to the expression levels of circCLK3 (Fig. 1c), we selected SiHa cells and HeLa cells to investigate the function and mechanism of circCLK3. To detect whether the head-to-tail splicing of circCLK3 results from trans-splicing or genomic rearrangements, cDNA and gDNA of SiHa cells were extracted. The gel electrophoresis results showed that circCLK3 was detected only in cDNA, but not in gDNA, (Fig. 1d) indicating that the loop structure of circCLK3 comes from reversely splicing. To identify the subcellular location of circCLK3 in cervical cancer cells, nuclear-cytoplasmic fractionation assays were performed. The results showed that most of circCLK3 was located in cytoplasm in SiHa and HeLa cells (Fig. 1e). In addition, RNA-FISH assays were also conducted with two cy3-labeled circCLK3 probes and indicated that most of circCLK3 was located in cytoplasm in SiHa and HeLa cells (Fig. 1f). To confirm the stability of circCLK3, SiHa, and HeLa cells were treated with actinomycin D, an inhibitor of RNA synthesis, and RNase R, a processive 3′ to 5′ exoribonuclease. qRT-PCR results showed that the half-life of circCLK3 exceeded 24 h in SiHa and HeLa cells, while that of CLK3 mRNA was <6 h (Fig. 1g), and that compared with linear mRNA CLK3, circCLK3 was more capable of resistance to digestion of RNase R (Fig. 1h), indicating that circCLK3 is highly stable in cervical cancer cells. These data indicated that circCLK3 is highly stable in cytoplasm of cervical cancer cells, implying its potential to be a promising diagnostic or prognostic biomarker.

We next detected the level of circCLK3 in 48 paired cervical cancer and adjacent normal tissues. qRT-PCR results showed that the expression level of circCLK3 was significantly higher in cervical cancer tissues than in adjacent normal tissues (Fig. 1i, *p* < 0.01). Based on the median expression of circCLK3, these samples were divided into two groups, high circCLK3 group and low circCLK3 group. Further statistical analyses indicated that increased expression of circCLK3 was significantly correlated with poor tumor differentiation (*p* = 0.015), advanced FIGO stages (*p* = 0.002), and large depth of stromal invasion (*p* = 0.008) (Table 1). Moreover, Kaplan–Meier Plotter analysis showed that cervical cancer patients with high circCLK3 had a shorter overall survival (Fig. 1j) and disease-free survival (Fig. 1k) than those with lower expression of circCLK3 (*p* < 0.01). These results suggest that circCLK3 is a highly stable circRNA and play a vital role in the progression of cervical cancer.

**Table 1:** Associations between the expression levels of circCLK3 and clinicopathological features in cervical cancer (*n* = 48).

Parameters	Category	No.	CircCLK3 expression		*χ*^*2*^	*P-*value
			High (%)	Low (%)		
Age					0.030	0.863
	<45	22	13	9		
	≥45	26	16	10		
Differentiation					5.976	0.015
	G1+G2	20	8	12		
	G3	28	21	7		
FIGO stage					9.261	0.002
	I	20	7	13		
	II	28	22	6		
Depth of stromal invasion					7.056	0.008
	<1/2	24	10	14		
	≥1/2	24	19	5		
Lymph node metastasis					1.024	0.312
	Yes	16	14	12		
	No	22	15	7		
Tumor size (cm)					0.426	0.514
	<4	25	14	11		
	≥4	23	15	8		

### CircCLK3 promotes cell proliferation, migration, and invasion of cervical cancer in vitro

**Fig. 2:**
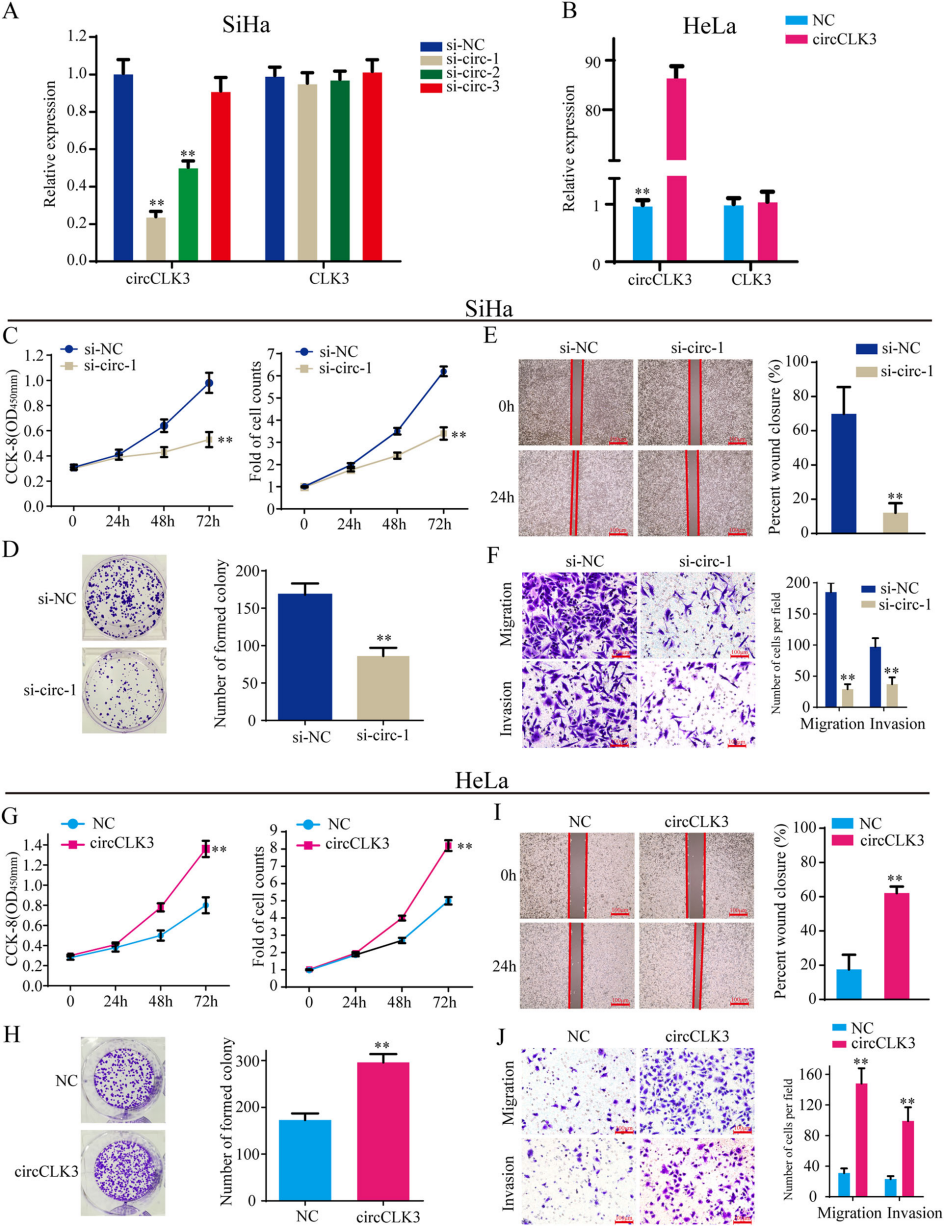
CircCLK3 promotes cell proliferation, migration, and invasion of cervical cancer cells in vitro. **a** qRT-PCR analysis of circCLK3 and CLK3 expression after transfection with three siRNAs targeted circCLK3 or control in SiHa cells. **b** qRT-PCR analysis of circCLK3 and CLK3 expression after transfection with pEX-3-circCLK3 or control in HeLa cells. **c** CCK-8 and cell count assays showed that knockdown of circCLK3 inhibited cell proliferation of SiHa cells. **d** Cell colony assay indicated that knockdown of circCLK3 inhibited cell colony formation of SiHa cells. **e**, **f** The wound healing and transwell assays showed that si-circCLK3 suppressed cell mobility, migration, and invasion of SiHa cells. **g** CCK-8 and cell count assays showed that overexpression of circCLK3 promoted cell proliferation of HeLa cells. **h** Cell colony assay indicated that overexpression of circCLK3 promoted cell colony formation of HeLa cells. **i**, **j** The wound healing and transwell assays showed that overexpression enhanced cell mobility, migration and invasion of HeLa cells. Data are reported as means ± standard deviation of three independent experiments. *p < 0.05; **p < 0.01.

To explore the possible biological function of circCLK3 in cervical cancer, three siRNAs (si-circ-1, si-circ-2, and si-circ-3) targeting the junction sites of circCLK3 and one overexpression vector (circCLK3) were generated. qRT-PCR analysis showed that both si-circ-1 and si-circ-2 obviously decreased the level of circCLK3, but not the level of CLK3, in SiHa cells (*p* < 0.01, Fig. 2a), and transfection of overexpression vector significantly increased the expression of circCLK3, but not the expression of CLK3, in HeLa cells (*p* < 0.01, Fig. 2b). We chose si-circ-1 for SiHa cells due to the highest inhibitory efficiency. CCK-8 and cell counts assays showed that knockdown of circCLK3 significantly decreased the ability of cell proliferation of SiHa cells (Fig. 2c). Cell colony assays confirmed the effects (Fig. 2d). In addition, wound healing assays, transwell migration and invasion assays showed that knockdown of circCLK3 suppressed the ability of mobility, migration and invasion in SiHa cells (Fig. 2e, f). Nevertheless, overexpression of circCLK3 enhanced cell proliferation (Fig. 2g, h), mobility (Fig. 2i), migration and invasion (Fig. 2j) in SiHa cells. Taken together, these results show that circCLK3 promotes cell proliferation, migration, and invasion of cervical cancer cells in vitro.

### CircCLK3 serves as a miRNA sponge for miR-320a in cervical cancer cells

**Fig. 3:**
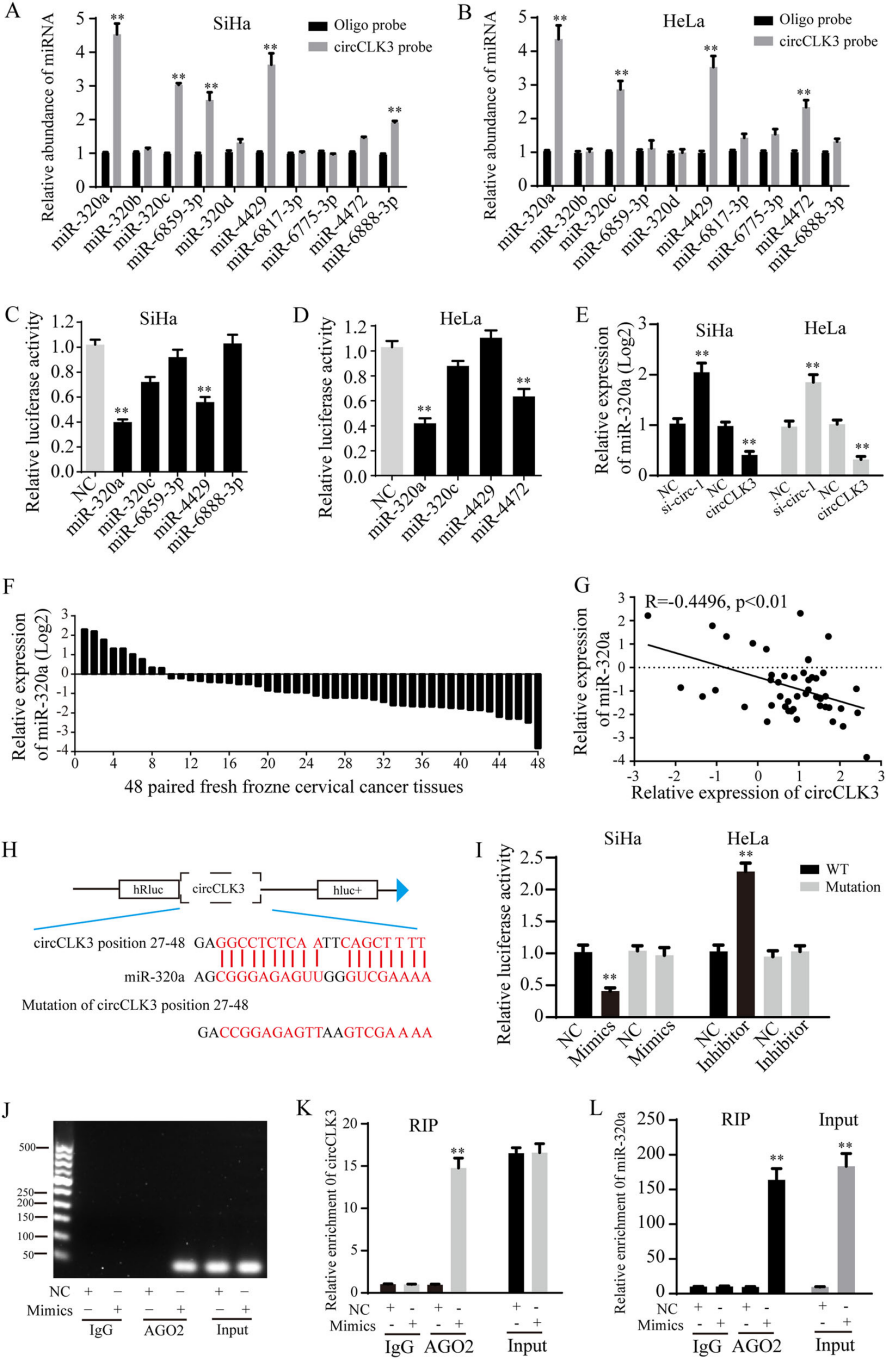
CircCLK3 acted as a ceRNA through directly binding to miR-320a. **a**, **b** The relative levels of 10 miRNA candidates in SiHa and HeLa lysates were examined by qRT-PCR. Multiple miRNAs were pulled-down by circCLK3 probe. **c**, **d** Relative luciferase activities were detected in cervical cancer cells after transfecting luciferase reporter plasmid of wild-type circCLK3 with selected miRNA mimics. **e** The effects of altered circCLK3 expression on miR-320a expression were examined by qRT-PCR. **f** MiR-320a was relatively low expressed in 81.25% (39/48) cervical cancer tissues. **g** Pearson’s correlation showed that miR-320a level negatively correlated with circCLK3 level in above 48 paired cervical cancer tissues. **h** Schematic representation of two potential binding sites of miR-320a on circCLK3 and both mutant binding sites. **i** The relative luciferase activity of mimics or inhibitor of miR-320a on the luciferase reporter plasmid with wild type circCLK3 sequence (WT) or both mutant binding sites of circCLK3 (Mutation) was detected in SiHa and HeLa cells. **j**, **k** An anti-AGO2 RIP assay was performed in SiHa cells after transfection with miR-320a mimics or negative control, followed by agarose gel electrophoresis (**j**) and qRT-PCR (**k**, **l**) to detect the expression of circCLK3 and miR-320a. Data are reported as means ± standard deviation of three independent experiments. *p < 0.05; **p < 0.01.

According to the hypothesis of ceRNA, circRNAs may exert biological functions by sponging miRNAs. Since circCLK3 is localized in cytoplasm, we speculated that circCLK3 could serve as a miRNA sponge. Then, the miRanda database was used to predict potential circRNA-miRNA interactions. The top 10 miRNAs with highest scores were selected. Then, a pull-down assay was performed with a biotinylated circCLK3 probe. The results showed that miR-320a, miR-320c, miR-6859-3p, miR-4429, and miR-6888-3p were captured in SiHa cells (Fig. 3a), while miR-320a, miR-320c, miR-4429, and miR-4472 were captured in HeLa cells (Fig. 3b). To confirm whether circCLK3 could sponge these miRNAs, a luciferase reporter plasmid with wild type of circCLK3 (WT) was constructed. Luciferase reporter assays showed that miR-320a mimics decreased the activities of luciferase reporter plasmid in both SiHa and HeLa cells (Fig. 3c, d). Thereby, we speculated that circCLK3 may act as a miR-320a sponge.

Furthermore, knockdown or overexpression of circCLK3 increased or suppressed miR-320a expression in SiHa and HeLa cells, respectively (Fig. 3e). Then, the expression levels of miR-320a in above 48 paired cervical cancer tissues were detected by qRT-PCR. Results showed that the level of miR-320a was low in most of cervical cancer tissues compared with adjacent normal tissues (Fig. 3f). Further analysis indicated that miR-320a level negatively correlated with circCLK3 level (Fig. 3g, *R* = −0.4496, *p* < 0.01). To further investigate the regulation of circCLK3 on miR-320a, a luciferase reporter plasmid (Mutation) with mutated binding sites of circCLK3 on miR-320a was constructed (Fig. 3h). Luciferase reporter assay results indicated that miR-320a mimics evidently decreased the luciferase activity of WT, but did not that of Mutation, while miR-320a inhibitor significantly elevated the luciferase activity of WT, but had no effect on that of Mutation (Fig. 3i). It has been widely known that miRNAs suppress translation or degrade mRNA of target genes in an AGO2-dependent manner. Therefore, an anti-AGO2 RNA immunoprecipitation (RIP) assay was used to immunoprecipitate circCLK3 by an anti-AGO2 antibody or control IgG. The results showed that both circCLK3 and miR-320a were significantly immunoprecipitated by anti-AGO2 antibody compared with IgG, and enriched by miR-320a mimics compared with negative control (Fig. 3j–l). These data demonstrated that circCLK3 acts as a ceRNA to sponge miR-320a by directly binding to MREs.

### MiR-320a suppresses FoxM1 expression

**Fig. 4:**
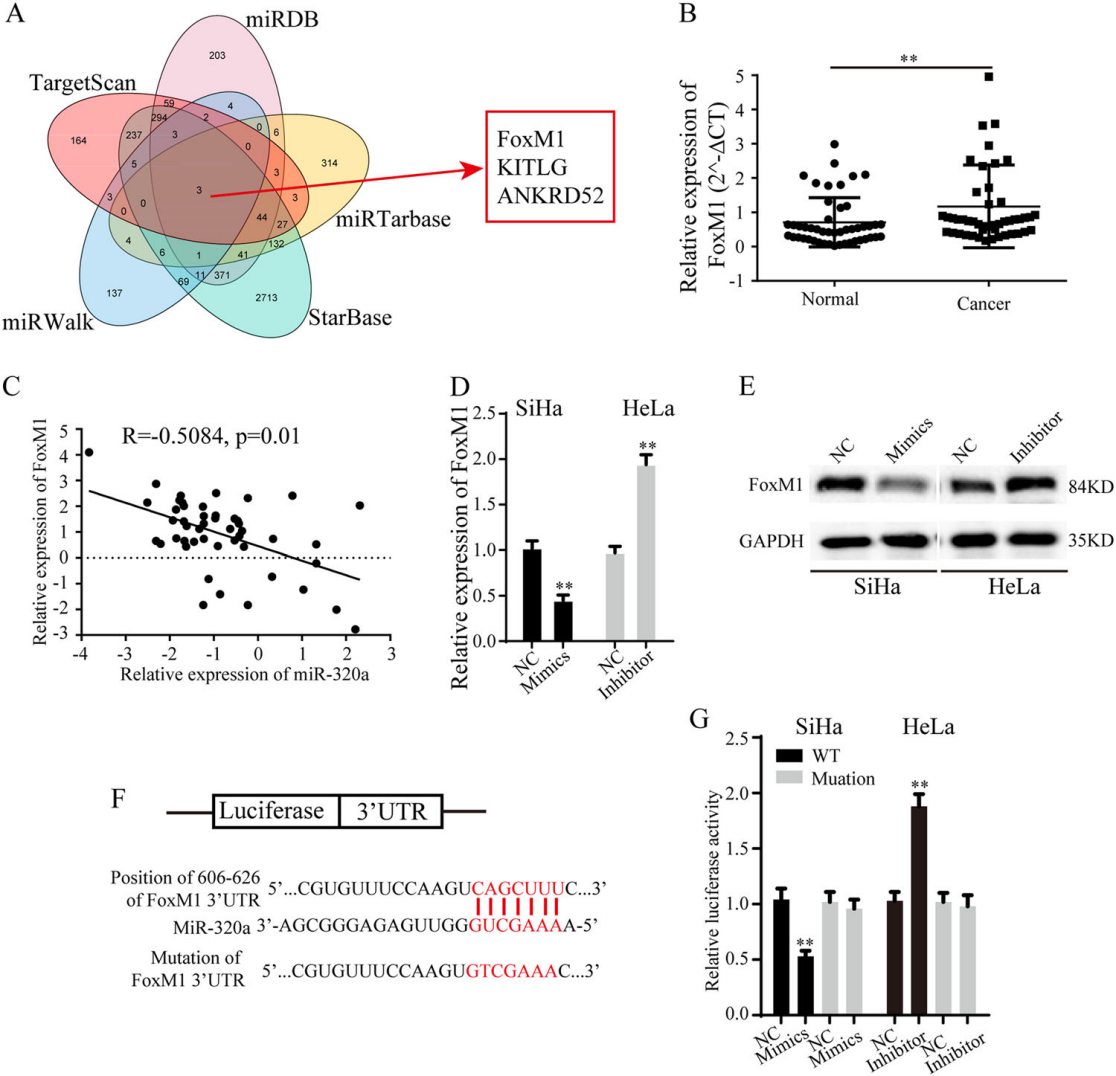
MiR-320a targets FoxM1 and suppresses its expression. **a** Bioinformatics databases were used to predict the potential target genes of miR-320a. **b** Relative expression of FoxM1 in above 48 paired cervical cancer tissues and adjacent normal tissues. **c** Pearson’s correlation between miR-320a level and FoxM1 level. **d**, **e** Relative FoxM1 mRNA (**d**) and protein (**e**) expression after treated with miR-320a mimics or inhibitor. **f** Schematic representation of potential binding sites of miR-320a on FoxM1 3′-UTR and mutant binding sites. **g** The effects of altered miR-320a expression on the relative luciferase activities of plasmids with wild type or mutant 3′-UTR of FoxM1 mRNA. Data are reported as means ± standard deviation of three independent experiments. *p < 0.05; **p < 0.01.

To explore the target gene of miR-320a, TargerScan, miRDB, miRTarbase, StarBase, and miRWalk databases were used to predict potential target genes. Three genes were found from the overlap between above 5 databases (Fig. 4a). As we know, FoxM1 has been widely reported to promote cell proliferation, invasion and EMT in multiple cancers. Then, we focused on FoxM1 for further exploration. qRT-PCR results showed that FoxM1 expression was significantly higher in above 48 paired cervical cancer tissues compared with adjacent normal tissues (Fig. 4b). Pearson’s correlation analysis indicated that the expression level of miR-320a negatively correlated with FoxM1 expression in these 48 paired cervical cancer tissues (Fig. 4c, *R* = −0.5084, *p* = 0.01), which implies that miR-320a may negatively regulate FoxM1 expression. Furthermore, miR-320a mimics down-regulated FoxM1 expression in SiHa cells, while miR-320a inhibitor caused increase of FoxM1 expression in HeLa cells (Fig. 4d, e). To explore the regulation mechanisms of miR-320a on FoxM1 expression, luciferase reporter plasmids with wild type of FoxM1 mRNA (WT) or mutant binding sites of FoxM1 mRNA on miR-320a (Mutation) were constructed (Fig. 4f). Luciferase reporter assays indicated that miR-320a mimics significantly decreased the luciferase activity of WT, but posed no effect on the luciferase activity of Mutation, while miR-320a inhibitor evidently increased the luciferase activity of WT, but did not increase that of Mutation (Fig. 4g). In summary, these data demonstrated that miR-320a negatively regulate FoxM1 expression by directly binding to 3′-UTR of FoxM1 mRNA.

### FoxM1 promotes cell proliferation, EMT, migration, and invasion of cervical cancer in vitro

**Fig. 5:**
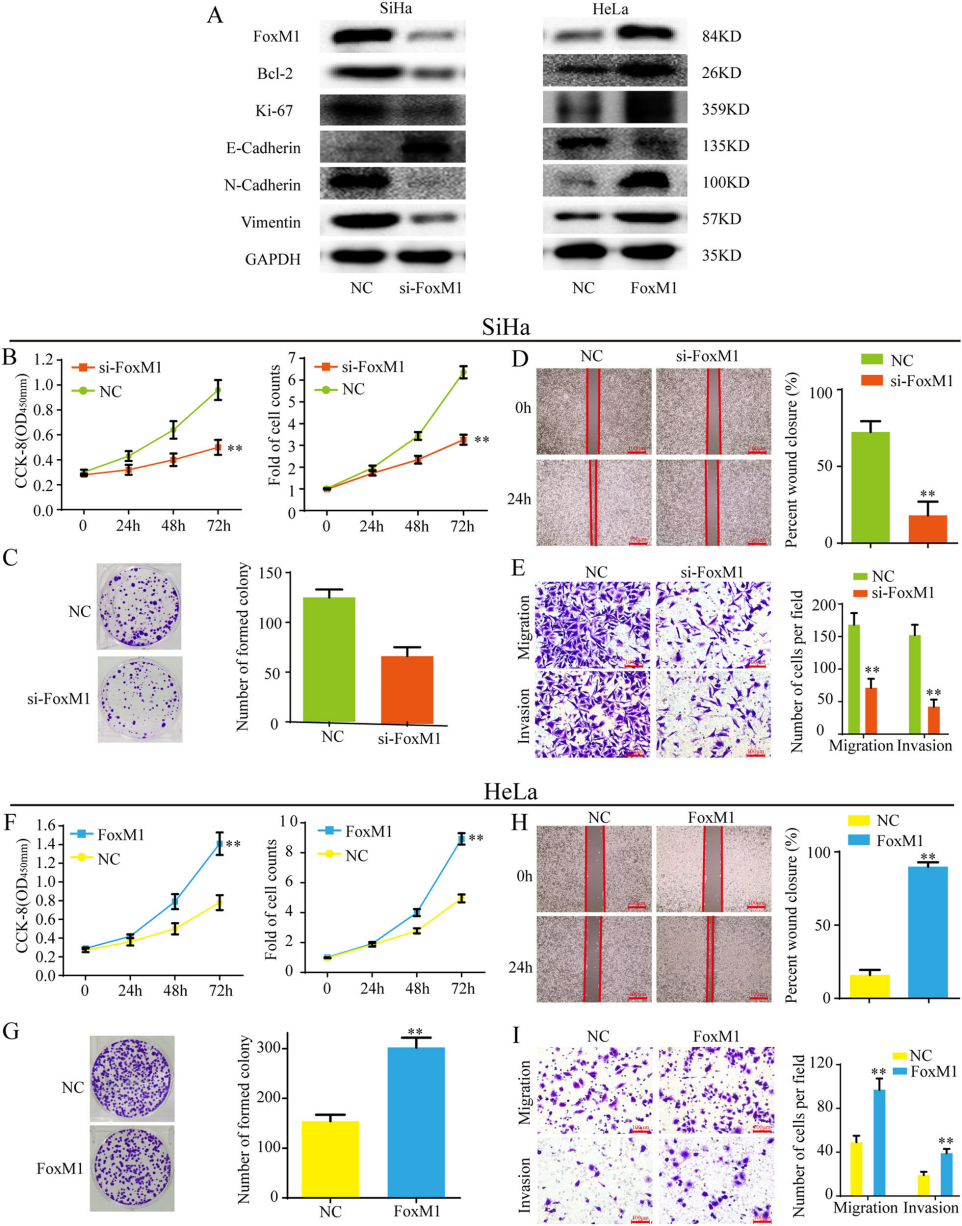
FoxM1 promotes cell proliferation, EMT, migration and invasion of cervical cancer cells in vitro. **a** si-FoxM1 inhibited the expression of proliferation-related proteins and EMT in SiHa cells. Overexpression of FoxM1 promoted the expression of proliferation-related proteins and EMT in HeLa cells. **b**, **c** CCK-8, cell count and cell colony assays showed that knockdown of FoxM1 inhibited cell proliferation of SiHa cells. **d**, **e** The wound healing and transwell assays showed that si-FoxM1 suppressed cell mobility, migration and invasion of SiHa cells. **f**, **g** CCK-8, cell count and cell colony assays showed that overexpression of FoxM1 promoted cell proliferation of HeLa cells. **h**, **i** The wound healing and transwell assays showed that overexpression of FoxM1 enhanced cell mobility, migration and invasion of HeLa cells. Data are reported as means ± standard deviation of three independent experiments. *p < 0.05; **p < 0.01.

In order to explore the biological functions of FoxM1 in cervical cancer, a si-RNA target FoxM1 and an overexpression vector of FoxM1 were generated. Western blot showed that si-FoxM1 significantly decreased the expression level of FoxM1 in SiHa cells, while the overexpression vector remarkably increased the level of FoxM1 in HeLa cells (Fig. 5a). Further, knockdown of FoxM1 evidently decreased the expression of cell proliferation-related proteins, such as Ki-67 and Bcl-2, while overexpression of FoxM1 increased the expression of Ki-67 and Bcl-2, indicating that FoxM1 promotes cell proliferation. Knockdown of FoxM1 significantly increased the expression of E-cadherin, the biomarker of epithelial, and decreased the expression of Vimentin and N-cadherin, the biomarker of mesenchymal, while overexpression of FoxM1 decreased E-cadherin expression, but increased Vimentin and N-cadherin expression, indicating that FoxM1 promotes EMT.

Functionally, CCK-8, cell counts, and cell colony assays showed that si-FoxM1 significantly decreased the ability of cell proliferation of SiHa cells (Fig. 5b, c), and that up-regulation of FoxM1 enhanced the ability of cell proliferation of HeLa cells (Fig. 5f, g). Wound healing assays, transwell migration and invasion assays showed that knockdown of FoxM1 suppressed the ability of mobility, migration, and invasion in SiHa cells (Fig. 5d, e), while overexpression of FoxM1 enhanced the ability of mobility, migration and invasion in HeLa cells (Fig. 5h, i). Taken together, FoxM1 promotes cell proliferation, EMT, migration, and invasion of cervical cancer cells.

### MiR-320a suppress cell proliferation, migration, and invasion through FoxM1

**Fig. 6:**
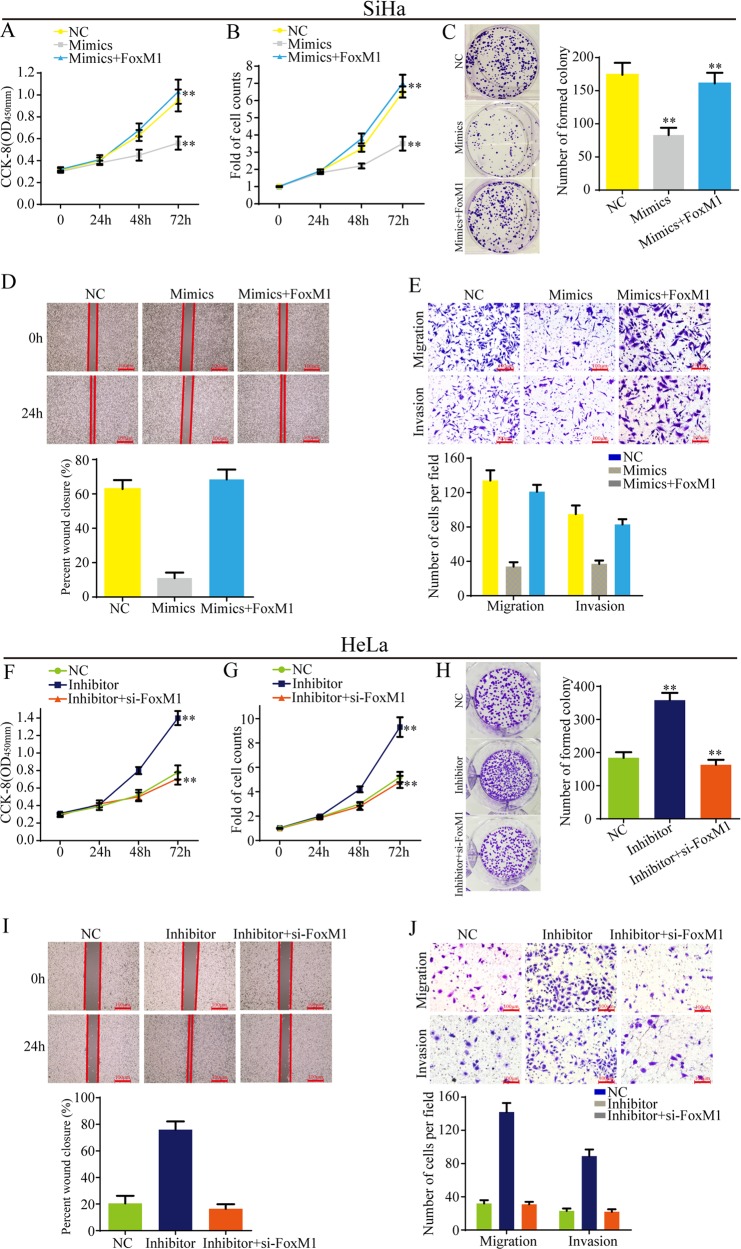
MR-320a suppresses cell proliferation, migration, and invasion through FoxM1. **a–c** CCK-8, cell count, and cell colony assays showed that miR-320a mimics inhibited cell proliferation. However, FoxM1 reversed the ability of miR-320a to inhibit cell proliferation. **d**, **e** The wound healing and transwell assays showed that miR-320a mimics suppressed cell mobility, migration, and invasion. However, FoxM1 reversed the ability of miR-320a to suppress cell mobility, migration, and invasion. **f–h** CCK-8, cell count, and cell colony assays showed that miR-320a inhibitor enhanced cell proliferation. However, si-FoxM1 suppressed the ability of miR-320a inhibitor to enhance cell proliferation. **i**, **j** The wound healing and transwell assays showed that miR-320a inhibitor promoted cell mobility, migration, and invasion. However, si-FoxM1 inhibited the ability of miR-320a inhibitor to promote cell mobility, migration, and invasion. Data are reported as means ± standard deviation of three independent experiments. *P < 0.05; **P < 0.01.

In this study, we investigated the biological functions of miR-320a in cervical cancer. The results showed that miR-320a mimics suppressed cell proliferation (Fig. 6a–c), migration, and invasion (Fig. 6d, e), while miR-320a inhibitor enhanced cell proliferation (Fig. 6f–h), migration, and invasion (Fig. 6i, j).

To further explore the effects of FoxM1 on the roles of miR-320a in cervical cancer progression, rescued experiments were conducted. As shown in Fig. 6a–j, overexpression of FoxM1 reversed the ability of miR-320a mimics to inhibit cell proliferation, migration, and invasion, while si-FoxM1 suppressed the ability of miR-320a inhibitor to promote cell proliferation, migration, and invasion. To summary, miR-320a suppresses cell proliferation, migration, and invasion through FoxM1.

### CircCLK3 promote progression of cervical cancer through FoxM1

**Fig. 7:**
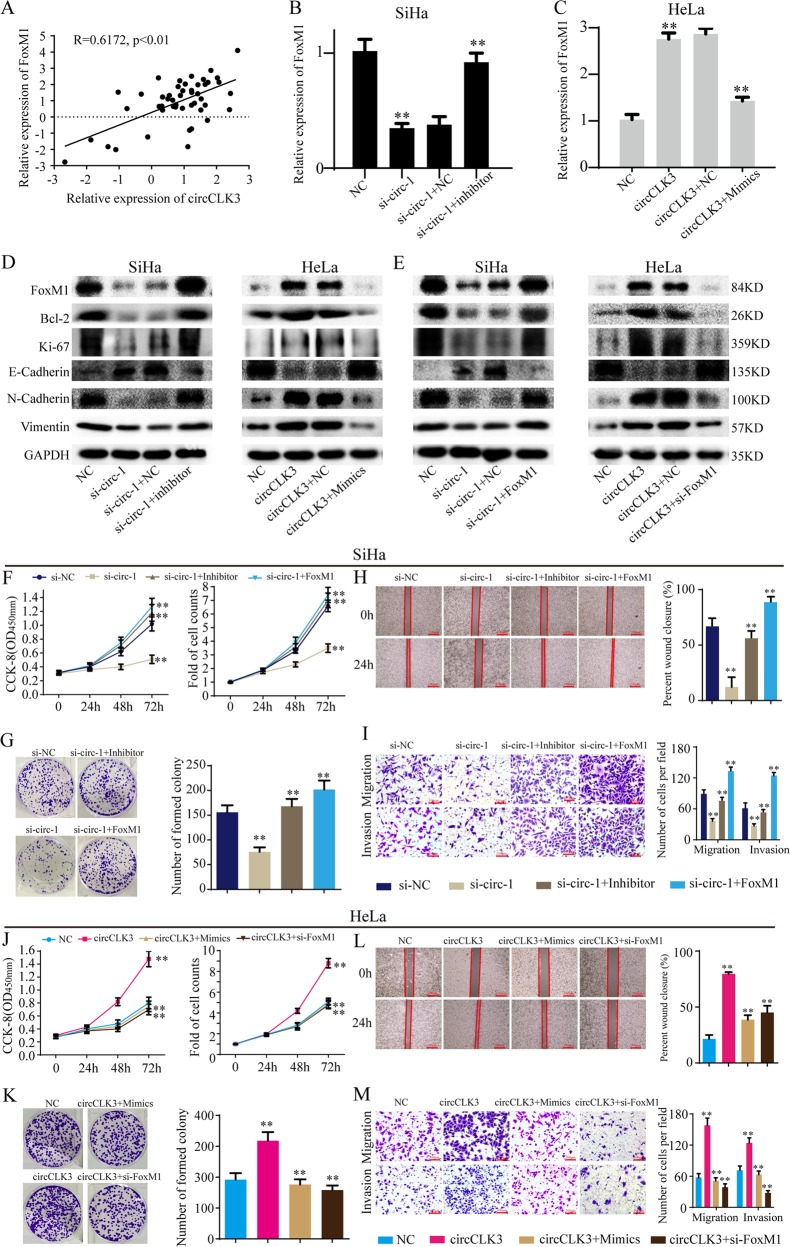
CircCLK3 promoted cervical cancer progression through miR-320a and FoxM1. **a** Pearson’s correlation between circCLK3 level and FoxM1 level in above 48 paired cervical cancer tissues. **b**, **c** The effects of altered circCLK3 and miR-320a expression on FoxM1 expression. **d** The effects of altered circCLK3 and miR-320a expression on FoxM1, proliferation-related and EMT-related proteins expression and EMT. **e** The effects of altered circCLK3 and FoxM1 expression on FoxM1, proliferation-related and EMT-related proteins expression and EMT. **f**, **g**, **j**, **k** The effects of altered circCLK3 and miR-320a or FoxM1 expression on cell proliferation. **h**, **i**, **l**, **m** The effects of altered circCLK3 and miR-320a or FoxM1 expression on the ability of mobility, migration, and invasion. Data are reported as means ± standard deviation of three independent experiments. *p < 0.05; **p < 0.01.

As a ceRNA, circCLK3 regulates target gene expression by sponging miRNAs. Therefore, we next explored whether circCLK3 regulates FoxM1 expression. Pearson’s correlation analysis showed that circCLK3 level positively correlated with FoxM1 level in 48 paired cervical cancer tissues (Fig. 7a). Knockdown of circCLK3 down-regulated FoxM1 mRNA and protein expression, and overexpression of circCLK3 up-regulated FoxM1 mRNA and protein expression (Fig. 7b–e). These results implied that circCLK3 promoted FoxM1 expression. In addition, si-circCLK3 decreased Bcl-2 and Ki-67 expression and overexpression of circCLK3 increased Bcl-2 and Ki-67 expression, indicating that circCLK3 promotes cell proliferation of cervical cancer (Fig. 7d, e). Also, si-circCLK3 increased E-cadherin expression and down-regulated Vimentin and N-cadherin expression, while overexpression of circCLK3 decreased E-cadherin expression and up-regulated Vimentin and N-cadherin expression, indicating that circCLK3 promotes EMT of cervical cancer (Fig. 7d, e). Furthermore, FoxM1 reversed the ability of si-circCLK3 to inhibit the expression of proliferation-related proteins and EMT, while si-FoxM1 suppressed the ability of circCLK3 to promote the expression of proliferation-related proteins and EMT (Fig. 7e).

Functionally, CCK-8, cell count, and cell colony assays showed that FoxM1 reversed the ability of si-circCLK3 to inhibit cell proliferation (Fig. 7f, g), and wound healing, transwell migration and invasion assays indicated that FoxM1 reversed the ability of si-circCLK3 to inhibit cell mobility, migration and invasion (Fig. 7h, i). Knockdown of FoxM1 suppressed the ability of circCLK3 to promote cell proliferation (Fig. 7j, k), mobility, migration, and invasion (Fig. 7l, m). Taken together, circCLK3 promotes cell proliferation, EMT, migration, and invasion through FoxM1.

### MiR-320a reverses the ability of circCLK3 to promote FoxM1 expression and progression of cervical cancer

To test the effects of miR-320a on the roles of circCLK3 to promote FoxM1 expression and progression of cervical cancer, rescue experiments were performed. MiR-320a inhibitor relieved the suppression of si-circCLK3 on FoxM1 mRNA and protein expression, while miR-320a mimics inhibited the promotion of circCLK3 on FoxM1 mRNA and protein expression (Fig. 7b–d). Furthermore, knockdown of miR-320a reversed the ability of si-circCLK3 to inhibit the expression of proliferation-related proteins and EMT, while overexpression of miR-320a suppressed the ability of circCLK3 to promote the expression of proliferation-related proteins and EMT (Fig. 7d). Functionally, CCK-8, cell count, and cell colony assays showed that miR-320a inhibitor reversed the ability of si-circCLK3 to inhibit cell proliferation (Fig. 7f, g), and wound healing, transwell migration, and invasion assays indicated that miR-320a inhibitor reversed the ability of si-circCLK3 to inhibit cell mobility, migration, and invasion (Fig. 7h, i). MiR-320a mimics suppressed the ability of circCLK3 to promote cell proliferation (Fig. 7j, k), mobility, migration, and invasion (Fig. 7l, m). Taken together, miR-320a reversed the ability of circCLK3 to promote the expression of FoxM1, cell proliferation, EMT, migration, and invasion in cervical cancer.

### CircCLK3 promotes growth and metastasis of cervical cancer cells in vivo

**Fig. 8:**
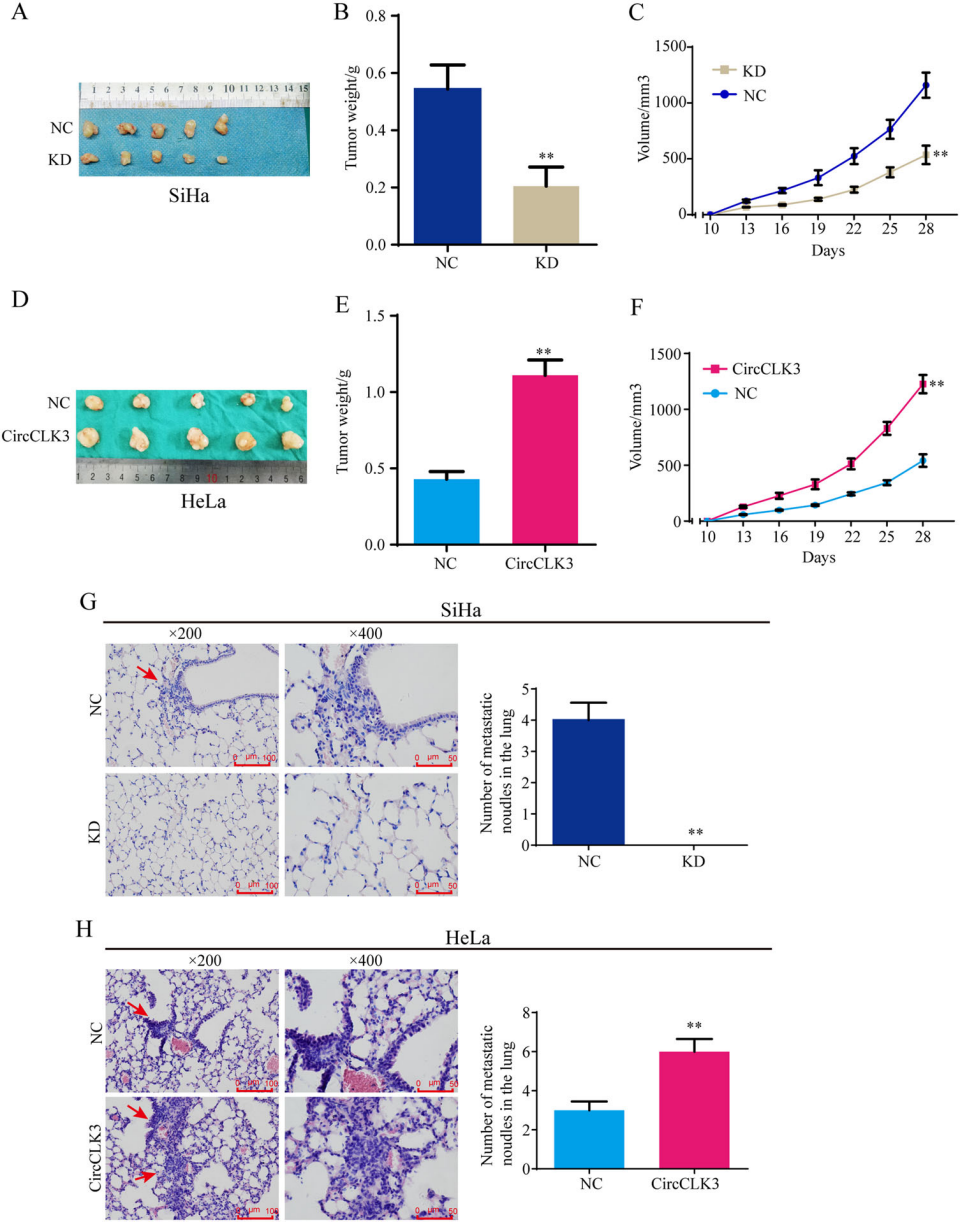
CircCLK3 promotes growth and metastasis of cervical cancer cells in vivo. **a**, **d** Represent pictures of tumor formation of xenograft of SiHa (**a**) and HeLa (**d**) cells in nude mice (*n* = 5). **b**, **e** Weight of tumors of SiHa (**b**) and HeLa (**e**) cells. **c**, **f** The tumor volume of SiHa (**c**) and HeLa (**f**) cells. The lung metastasis models were constructed. **g**, **h** Representative images of HE staining of the lung. Knockdown of circCLK3 decreased the number of lung metastatic nodules compared with negative control (**g**), while overexpression of circCLK3 increased the number of lung metastatic nodules compared with negative control (**h**). *p < 0.05; **p < 0.01.

To determine whether circCLK3 affects tumor growth in vivo, xenograft mouse models were established by subcutaneously injecting equal amounts of cervical cancer cells (*n* = 5 for each group). After 28 days, all mice were killed and tumor samples were harvested (Fig. 3a, d). As shown in Fig. 8, the weight (Fig. 8b) and volume (Fig. 8c) of tumors with knockdown of circCLK3 were significantly lower than those with control in SiHa cells (*p* < 0.01 for both). Likewise, the weight (Fig. 8e) and volume (Fig. 8f) of tumors with overexpression of circCLK3 were both significantly higher than the control tumors of HeLa cells (*p* < 0.01 for both).

The role of circCLK3 on metastasis was confirmed by in vivo lung metastasis models. In the lung metastasis models, necropsy and HE staining showed that the number of lung metastatic nodules was less in mice with knockdown of circCLK3 compared with negative control (Fig. 8g), while the number was higher in mice with overexpression of circCLK3 than with negative control (Fig. 8h). Overall, these data demonstrate that circCLK3 promotes growth and metastasis of cervical cancer in vivo.

## Discussion

Up to now, large amounts of miRNAs and lncRNAs have been reported to play large roles in the development and progression of cervical cancer. However, the biological functions of circRNAs in cervical cancer are rarely covered. In this study, we found that a novel circRNA, circCLK3 promoted cell proliferation, EMT, migration, and invasion in cervical cancer. Mechanistically, circCLK3 acted as a ceRNA to sponge miR-320a, and thus relieved its ability to suppress the expression of FoxM1.

As a new class of endogenous non-coding RNAs, circRNA is gradually entering the field of tumor research^31^. Identifying ectopicly expressed circRNAs and determining their roles in cancers has been attracting attention. For example, Yang et al. found that circ-ITCH was down-regulated in bladder cancer tissues, and inhibited cell proliferation, invasion and metastasis both in vitro and in vivo^32^. CircIRAK3 has been reported to play an oncogenic role and promoted cell migration, invasion, and metastasis in breast cancer, and may be a potential biomarker for metastasis and target for therapy^33^. Additionally, high circHIPK3 expression was positively correlated with metastasis, advanced clinical stages and poor overall survival, and knockdown of circHIPK3 inhibited cell proliferation and invasion, induced cell apoptosis, suggesting its roles as an oncogene in colorectal cancer.^34^ However, few circRNAs has been reported to participate in the development and progression of cervical cancer at present^35^. In our study, differentially expressed circRNAs were screened between three paired cervical cancer tissues and adjacent normal tissues. CircCLK3 expression was found to be significantly higher in cervical cancer tissues, which was also demonstrated by qRT-PCR results in cervical cancer tissues. Further analysis showed that high expression of circCLK3 was positively correlated with poor tumor differentiation, advanced FIGO stages and large depth of stromal invasion; moreover, high expression of circCLK3 predicted a short overall survival and disease-free survival, suggesting that circCLK3 may serve as a predictor for survival of patients with cervical cancer. In addition, circCLK3 is more stable in cells than its corresponding linear mRNA; thus, circCLK3 can be used as a biomarker for diagnosis and target for treatment. In functional experiments, circCLK3 was demonstrated to promote cell growth and metastasis in vitro and in vivo. These results suggest that circCLK3 acts as an oncogenic factor to promote the progression of cervical cancer, and may well be a promising biomarker for diagnosing, a predictor for survival and a target for treatment. However, whether circCLK3 can be detected in blood plasma, circulating tumor cells and exosomes is an interesting and profound issue to be further explored.

CircRNAs generate from exonic, intronic or intergenic regions, and possess exclusive sequences from their parental transcripts^36–38^, suggesting that circRNAs have the potential to bind to the same miRNAs with their parental transcripts. Accumulating evidences indicated that circRNAs are confirmed to act as a ceRNA to sponge miRNAs and thereby to protect target genes from being inhibited or degraded by miRNAs^39–42^. Zhong et al. found that circRNA-MYLK competitively binds to miR-29a and relieves the suppression of target gene VEGFA, which then activated VEGFA/VEGFR2 signaling pathway^43^. Han et al. detected that circMTO1 expression was significantly down-regulated in hepatocellular carcinoma tissues, and low circMTO1 expression was closely correlated with shorter survival^44^. Furthermore, they clarified that circMTO1 acted as the sponge of miR-9 to promote p21 expression, and thus suppressed cell proliferation and invasion. In addition, Hsiao et al. verified that a novel circRNA, circCCDC66 was elevated in colon cancer tissues compared with adjacent normal tissues, and promoted cell proliferation, migration and invasion by acting as a sponge of miR-33b and miR-93 to release the inhibition of MYC^45^. Our data support the findings that circCLK3 acted as a ceRNA to sponge miR-320a. First, most of circCLK3 was located in cytoplasm, demonstrating that circCLK3 plays roles on transcriptional or post-transcriptional level. Second, circCLK3 level was negatively correlated with miR-320a level in cervical cancer tissues. Third, down-regulated circCLK3 caused increased miR-320a expression, while up-regulated circCLK3 resulted in decreased miR-320a expression. Fourth, bioinformatics analysis found that circCLK3 shares the MREs of miR-320a. Fifth, pull-down assay demonstrated that miR-320a was pulled-down by a biotinylated circCLK3 probe, indicating the interaction between circCLK3 and miR-320a. Sixth, RIP assay indicated that circCLK3 recruits and directly binds to miR-320a in an AGO2 independent manner. Finally, luciferase reporter assay demonstrated that miR-320a regulate the luciferase activity of luciferase reporter plasmids with circCLK3, demonstrating the direct regulation of circCLK3 with miR-320a.

MiRNAs have been shown to regulate multiple of target genes in the development and progression of various cancers, by binding to MREs in the 3′-UTR of target genes^46,47^. Yu et al. found that miR-182 potentiates TGFβ-induced EMT, and promotes invasion of breast cancer cells and osteoclastogenesis for bone metastasis^48^. In terms of mechanism, they determined that miR-182 directly binds to the 3′-UTR of SMAD7 mRNA, which plays key roles in TGFβdownstream pathway. Long et al. reported that overexpressed miR-92-3p suppressed the proliferation and invasion of pancreatic cancer cells by suppressing the expression of Gabra3^49^. However, the biological functions and underlying molecular mechanisms of miR-320a in the development of cervical cancer remain elusive. As a transcription factor, FoxM1 has been reported to promote cell proliferation, migration, invasion and EMT in various cancers. In this study, a series of molecular experiments demonstrated that miR-320a suppresses FoxM1 expression through directly binding to 3′-UTR of FoxM1 mRNA. Functionally, miR-320a inhibits cell proliferation, EMT, migration and invasion in cervical cancer. However, FoxM1 reverses the ability of miR-320a to inhibit cell proliferation, EMT, migration, and invasion. These results indicated that miR-320a suppresses cell proliferation, EMT, migration, and invasion of cervical cancer cells by inhibiting FoxM1 expression.

To further explain the mechanisms of circCLK3 in the progression of cervical cancer, rescue experiments were performed. CircCLK3 promotes cell proliferation, EMT, migration, and invasion through FoxM1, while miR-320a reverses the ability of circCLK3 to promote progression of cervical cancer. In summary, circCLK3 acts as a miR-320a sponge to relieve its suppression on FoxM1 expression, and thereby promotes cell proliferation, EMT, migration, and invasion of cervical cancer. However, one circRNA may share multiple miRNA binding sites, while one miRNA can also bind to different circRNAs. Hence, circRNAs may interact with many different miRNAs in different biological processes. Accordingly, more studies are needed to reveal the functions and mechanisms of circCLK3 in cancers.

In conclusion, circCLK3 is first demonstrated to be a candidate oncogene in cervical cancer and closely correlated with clinicopathological features and survival. CircCLK3 acts as a ceRNA of miR-320a to abolish its suppressive effects on target gene FoxM1, and thereby promotes cell proliferation, EMT, migration and invasion of cervical cancer. Our findings suggest that the circCLK3/miR-320a/FoxM1 axis plays a key role in the progression of cervical cancer, and may well be a biomarker for diagnosis and target for therapy.

## Materials and methods

### Patients and clinical specimens

Forty-eight pairs of fresh frozen cervical cancer tissues and adjacent normal tissues were collected from patients after surgical resection at The Third Affiliated Hospital of Zhengzhou University from 2013 to 2018. None of the patients has undergone radiotherapy or chemotherapy before surgery. The clinicopathological diagnosis was determined by two pathologists according to the guidelines of International Federation of Gynecology and Obstetrics (FIGO). The project was approved by Institutional Review Boards of The Third Affiliated Hospital of Zhengzhou University. Informed consent was signed by all subjects before enrollment in the research.

### Cell lines and culture conditions

The human cervical cancer cell lines (SiHa, HeLa, CaSki, C-33A, MS751) were purchased from the Type Culture Collection of the Chinese Academy of Science (Shanghai, China). All cell lines were cultured in high glucose DMEM (HyClone) with 10% fetal bovine serum (FBS) (Gibco, Australia) and 1% penicillin-streptomycin at 37 °C in a humidified atmosphere containing 5% CO_2_.

### Oligonucleotides, plasmids, and transfection

Oligonucleotides and plasmids of circCLK3, miR-320a, and FoxM1 were designed and synthesized. The full length of circCLK3 was cloned into pEX-3 vector (Shanghai GenePharma CO. Ltd). Three siRNAs target circCLK3 were synthesized by RiBoBio (Guangzhou, China) as follows: si-circ-1, target: 5′-GGCCTTGTGTGAGCCACCA-3′; si-circ-2, target: 5′-ATAGGCCTTGTGTGAGCCA-3′; si-circ-3, target: 5′-GCCTTGTGTGAGCCACCAT-3′. The mimics and inhibitor of miR-320a were purchased from RiBoBio. A si-FOXM1 target: CTCTTCTCCCTCAGATATAdTdT. The oligonucleotides and plasmids were transfected into cells by LipofectamineTM 2000 (Invitrogen, Thermo Fisher Scientific, USA) according to the manufacturer’s instructions.

### RNA extraction and quantitative real-time PCR

Total RNA was extracted from cells and tissues using TRIzol (TaKaRa, Shiga, Japan) according to manufacturer’s instructions. RNA was reversely transcribed into cDNA using PrimeScript RT Reagent Kit (TaKaRa, Shiga, Japan). RNA expression was quantified by qRT-PCR with SYBR Premix Ex Taq™ (TaKaRa, Shiga, Japan). GAPDH or U6 were adapted as internal controls for circRNA, mRNA or miRNAs. The ΔΔCt method was employed to assay relative expression levels. The primers for circCLK3 are as follows: F: 5′-TGCATAAAATAGGCCTTGTGTGAG-3′, R: 5′-AGGCAGATTCACAGCCTCACTC-3′. The primers for FoxM1 are as follows: F: 5′-ACGTCCCCAAGCCAGGCTC-3′, R: 5′-CTATGTAGCTCAGGAATAA-3′. The primers for GAPDH are as follows: F: 5′-CACCATTGGCAATGAGCGGTTC-3′, R: 5′-AGGTCTTTGCGGATGTCCACGT-3′.

### Nuclear-cytoplasmic fractionation

Nuclear and cytoplasmic RNA was isolated with PARIS™ Kit (Invitrogen, USA) following the manufacturer’s instruction. In all, 1.0 × 10^7^ cells were collected and washed with PBS, then resuspended with 500 μl cell fractionation buffer. After incubating on ice for 10 min, the lysate was centrifuged for 5 min at 4 °C to separate nuclear and cytoplasmic RNA fractions. Cytoplasmic RNA fraction in the supernatant was transferred to an RNase-free tube. Nuclear pellet in precipitation was lysed with 500 μl cell disruption buffer. Mix the above Cytoplasmic RNA lysate or Nuclear pellet lysate with an equal volume of 2 × Lysis/Binding solution, then add an equal volume of 100% ethanol. At last, nuclear and cytoplasmic RNA fractions were draw through a filter cartridge. After washing and eluting, RNAs were stored at −80 °C for further detecting.

### RNA-Fluorescence in situ hybridization

RNA-Fluorescence in situ hybridization (RNA-FISH) assay was performed to identify the subcellular location of circCLK3 in cervical cells. Two Cy3-labeled circCLK3 probes were synthesized by GenePharma (Shanghai, China). The sequences of Cy3-labeled circCLK3 probes were as follows: 1. 5′-TGGGCATGGTGGCTCACACACAAGG-3′; 2. 5′-GGGCATGGTGGCTCACACACAAGGC-3′. Hybridization was performed overnight with circCLK3 probes according to the manufacturer’s instructions. The images with the magnification of 400 were acquired on fluorescence microscope.

### Western blotting

Total protein was extracted from cells using Radio-Immunoprecipitation Assay (RIPA) with 1% protease inhibitor phenylmethanesulfonyl fluoride (Beyotime Biotechnology, Jiangsu, China). The protein concentrations were detected by BCA Protein Assay Kit (Beyotime Biotechnology, Jiangsu, China) according to the manufacturer’s instructions. Electrophoresis was performed with sodium dodecyl sulfate-polyacrylamide gel electrophoresis (SDS-PAGE), and protein was transferred onto polyvinylidene fluoride (PVDF) membranes (Millipore, MA, USA). Then, the membranes were blocked in 5% non-fat milk at room temperature for 1.5 h, followed by incubating with the appropriate primary antibody at 4 °C overnight, and the secondary antibodies at room temperature for 1.5 h. Finally, the protein was developed by electrochemiluminescence (ECL) reagent (Millipore, MA, USA). The primary antibodies were as follows: FoxM1 (1:1000, Santa Cruz, USA), Bcl-2 (1:10,000, Abcam, USA), Ki-67 (1:10,000, Abcam, USA), E-Cadherin (1:10,000, Abcam, USA), N-Cadherin (1:5000, Abcam, USA), Vimentin (1:1000, Abcam, USA), GAPDH (1:1000; Cell Signaling Technology, USA).

### RNase R and actinomycin D treatment

Total RNA (10 μg) was incubated for 1 h at 37 °C with 40 U RNase R (Epicentre Technologies, Madison, USA) to degrade linear RNAs. Actinomycin D (2 mg/ml) or DMSO (Sigma-Aldrich, USA) was added into the cell culture medium to assay the stability of RNAs. After treatment with RNase R or actinomycin D, the RNA expression levels of circCLK3 and CLK3 were detected by qRT-PCR.

### CCK-8, cell count, and cell colony assay

For CCK-8 and cell count assays, cells were seeded into 96-well plates with 2000 cells per well and then cultured at 37 °C in a humidified atmosphere with 5% CO2. After culturing for 0, 24, 48, and 72 h, the optical density of each well was measured with CCK-4 kit (Beyotime Biotechnology, Jiangsu, China), and the cell numbers were counted with a Celigo® Image Cytometer (Nexcelom, USA).

For cell colony assay, cells were seeded into 6-well plates with 500 cells per well. After two weeks, the cells were fixed with 75% ethanol, then stained with 0.1% crystalline purple. Finally, the cells were photographed and counted.

### Cell wound healing and transwell assay

Cells were cultured to full confluence in 6-well plates. Then, cells were gently scratched with a 20-μl micropipette tip in the center of the well, followed by incubating with serum-free medium. Images were captured at 0 and 24 h after scratching. The width of wound healing was quantified and compared with baseline values. All experiments were independently repeated in triplicate.

For transwell assay, 2 × 10^4^ cell of each group suspended in 200 μl serum-free medium was plated in the upper chamber (8.0 μm pore, Corning, USA) with (invasion) or without (migration) Matrigel (BD Bioscience, USA), and 600 μl DMEM medium with 10% FBS was added to the bottom chamber. After incubating for 24 h, the chambers were fixed with 4% polymethanol and then stained with 0.1% crystal violet. The cells that migrated or invaded to the reverse side of the chambers were photographed and calculated. Five random fields were selected to calculate cells that migrated or invaded.

### Luciferase reporter assay

In this assay, SiHa and HeLa cells were seeded in 24-well plates. Luciferase reporter plasmids were co-transfected with mimics or inhibitor of miR-320a using LipofectamineTM 2000 reagent. After 36 h, cells were lysed with 100 μl 1 × passive lysis buffer (PLB), and rocked for 15 min at room temperature. The activities of firefly luciferase and Renilla luciferase were detected with Dual-luciferase reporter Assay Kit (Promega, USA). The relative activity was equal to firefly luciferase activity/Renilla luciferase activity. All experiments were independently repeated in triplicate.

### RNA immunoprecipitation assay

RIP was conducted in SiHa cells with magna RIPTM RNA-binding Protein Immunoprecipitation kit (Millipore, Billerica, MA). SiHa cells were transfected with miR-320a mimics, and then lysed in complete RNA lysis buffer after 48 h. Cell lysates were rotated in RIP immunoprecipitation buffer including magnetic beads conjugated with negative control mouse IgG or human anti-AGO2 antibody (Mouse, Millipore, Billerica, USA) overnight. Next day, immunoprecipitated RNA was extracted after incubating with Proteinase K for 30 min. Last, qRT-PCR and agarose gel electrophoresis were performed to identify the expression of circCLK3 and miR-320a.

### Pull-down assay

The biotinylated circCLK3 probe was synthesized by RiboBio (Guangzhou, China). Briefly, for pull-down assay, 1 × 10^7^ SiHa and HeLa cells were collected and lysed with ultrasonic. Probe-coated beads were generated by incubating oligo probe or circCLK3 probe with C-1 magnetic beads (Life Technologies) at 25 °C for 2 h. Then, the cell lysates were incubated with oligo probe or circCLK3 probe at 4 °C overnight. After washing with wash buffer, the RNA complexes were extracted with the RNeasy Mini Kit (QIAGEN, Germany). Finally, qRT-PCR was performed to identify the expression of selected miRNAs.

### Animal experiments

20 male BALB/c athymic nud mice (4 weeks old) were randomly divided into four groups (*n* = 5). To establish xenograft mouse models, the shRNA against circCLK3 (the same target with si-circ-1) were cloned into pLL3.7 vector and the full-length of circCLK3 were cloned into PLCDH-ciR vector, containing a front and back circular frame. Then, the stable cell lines were constructed with SiHa or HeLa cells. SiHa or HeLa cells were subcutaneously injected into left inguinal region with 1.0 × 10^7^ cells in 100 μl PBS. Tumor volumes were calculated by the formula: volume = (length × width^2^)/2. Finally, the mice were killed, and the volume and weight of tumors were detected.

To explore the effects of circCLK3 on metastasis of cervical cancer, we established the mouse model of lung metastasis. In all, 1.0 × 10^7^ stable cells were intravenously injected into the tail vein of nude mice. After 4 weeks, all mice were killed. The lungs were removed and validated by hematoxylin and eosin (H&E) staining. The animal experiments were approved by the Institutional Animal Care and Use Committee of The Third Affiliated Hospital of Zhengzhou University, and were performed according to the guidelines for the care and use of laboratory animals.
